# Study Protocol: It is time to dig deeper: A cross-country implementation mapping study of the iFightDepression® (online self-management) tool

**DOI:** 10.1371/journal.pone.0343982

**Published:** 2026-03-12

**Authors:** Thomas Rotter, Milica Petrovic, Mikołaj Zarzycki, Caroline Allenhof, Katharina Schnitzspahn, Piotr Toczyski, Azucena Justicia, Saiko Allende Leal, James Chan, Johannes Leimhofer, Katharina Scholze, Stefan Hackel, Victor Pérez Sola, Ulrich Hegerl

**Affiliations:** 1 School of Nursing, Queen’s University, 92 Barrie Street, Kingston, Ontario, Canada; 2 Research Center of the German Foundation for Depression and Suicide Prevention, Department of Psychiatry, Psychosomatics and Psychotherapy, University Clinic, Goethe University, Heinrich-Hoffmann-Str 10, Frankfurt, Germany; 3 School of Psychology and Sport Science, College of Medicine and Health, Bangor University, Bangor, Wales, United Kingdom & Betsi Cadwaladr University Health Board, NHS Wales, Wales, United Kingdom; 4 German Foundation for Depression and Suicide Prevention, Goerdelerring 9, Leipzig, Germany; 5 European Alliance Against Depression (EAAD) e.V., Goerdelerring 9, Leipzig, Germany; 6 Department of Media Sociology and Social Communication, The Maria Grzegorzewska University, Warsaw, Poland; 7 Hospital del Mar Research Institute, CIBER of Mental Health, Instituto de Salud Carlos III, Hospital del Mar, Barcelona, Spain; 8 Centre for Biomedical Research in Mental Health (CIBERSAM), Barcelona, Spain; 9 Chief Executive Officer (CEO) Sault Ste. Marie YMCA, Ontario, Canada; 10 Centre for Biomedical Research in Mental Health (CIBERSAM), Spain; Department of Psychiatry and Forensic Medicine, Universitat Autònoma de Barcelona, Barcelona, Spain; 11 Institut de Neuropsiquiatria i Addiccions, Parc de Salut Mar, Universitat Pompeu Fabra, Barcelona, Spain; 12 Goethe Research Professorship, Department of Psychiatry, Psychosomatics and Psychotherapy, University Hospital, Goethe University, Heinrich-Hoffmann-Straße 10, Frankfurt am Main, Germany; PLOS: Public Library of Science, UNITED KINGDOM OF GREAT BRITAIN AND NORTHERN IRELAND

## Abstract

**Background:**

The iFightDepression® (iFD) tool is an internet-based self-management program for individuals with milder forms of depression, used alongside support from trained user guides (e.g., family physicians). Despite strong evidence supporting guided internet-based cognitive behavioural therapy (iCBT), adherence to the iFD tool and its uptake across implementing countries remain variable.

**Methods:**

This implementation mapping study will be conducted in Germany, Poland, and Spain. Guided by the Consolidated Framework for Implementation Research (CFIR), we aim to identify barriers, facilitators, and country-specific implementation strategies. In Phase 1, we will invite 15 trained iFD user guides to complete an online qualitative survey to explore user experiences and perceived influences on patient uptake and adherence. In Phase 2, three country-specific focus groups will be conducted with trained user guides (one in each country). Survey data will be analyzed using Interpretative Phenomenological Analysis (IPA) and focus group data will be analyzed using template analysis.

**Discussion:**

Findings will be used to develop an iFD implementation manual and country-specific implementation blueprints. These outputs are intended to support consistent and sustainable adoption of this internet-based intervention across participating countries and to improve patient uptake and adherence.

**Study registration:**

The study, including the analysis plan, has been preregistered via the Open Science Framework (internet archive link: https://archive.org/details/osf-registrations-wmbhy-v1) following ethical approval.

## Background

Common mental health conditions such as depressive and anxiety disorders are highly prevalent and contribute substantially to the global burden of disease. More than 300 million people worldwide are estimated to suffer from depression, representing approximately 4.4% of the global population [1–3]. Although effective evidence-based treatments exist, access to care remains a pressing challenge in many countries, including across Europe.

Over recent decades, a wide range of clinical interventions have been developed to help improve access to mental health services [4]. iCBT has emerged as an effective treatment for common mental disorders and has demonstrated clinical outcomes comparable to face-to-face psychotherapy [5–13]. Evidence also indicates that guided iCBT, where a trained healthcare professional provides support during treatment, enhances adherence and leads to greater symptom reduction compared to unguided internet-based interventions [11,14–16].

Despite this evidence, however, routine uptake and adherence to iCBT programs remain limited [17,18]. Adherence rates in real-world settings are reported to range from approximately 12% for unguided interventions to 22% for guided interventions [11]. These low adherence rates highlight the need to better understand why many patients who are referred to an iCBT service either do not initiate treatment or discontinue early.

### The iFightDepression®-tool (Intervention)

The iFightDepression® (iFD) tool [19] is an internet-based self-management programme for individuals experiencing milder forms of depression. Versions exist for youth (15–24 years) and adults (25 + years). The tool is currently available in 15 languages and is intended to help individuals self-manage their symptoms of depression and promote recovery. It is based on Cognitive Behavioural Therapy (CBT) and contains six core and three optional workshops, including worksheets and exercises, as well as a mood-screening tool based on the Patient Health Questionnaire [20].

Through a training program offered either in person or online, user guides, such as family physicians, are trained in both depression management and the use of the iFD tool to support patients who may benefit from it. The iFD tool was primarily developed for family physicians as user guides; however, other licensed healthcare professionals qualified to treat depression according to national medical guidelines, such as psychiatrists and psychotherapists, can also become iFD user guides. Guidance refers to encouraging users and providing support when questions arise or when difficulties with the material are encountered. The iFD tool is available free of charge to both patients and guides [19].

### Rationale

Despite a strong evidence base supporting the use of the iFD tool for individuals with mild to moderate depression [10,11,14–16], adherence to the iFD tool remains lower than anticipated [11], with uneven uptake observed across countries that have implemented this internet-based depression programme [21]. Maximizing the iFD tool’s use is essential; otherwise, it’s benefits and effectiveness remain limited. Greater understanding of the barriers to and facilitators of adherence is therefore needed to inform tailored interventions for patients at increased risk of non-adherence [21].

This implementation study has the potential to enhance our understanding of the implementation process and may increase the uptake and adherence to guided iCBT interventions.

## Study design

### Theoretical framework

In this study, we are using the Consolidated Framework for Implementation Research (CFIR) as the guiding theoretical framework for this implementation mapping study [22–24]. CFIR is a conceptual framework that allows a comprehensive examination of factors that affect the implementation of the iFD tool at multiple levels. It considers 39 constructs in five areas or domains [23]. The domains are Intervention Characteristics, Inner Setting, Outer Setting, Characteristics of Individuals, and Implementation Process.

As a determinant framework, it has informed the development of our focus group interview questions, and it will guide the development of country-specific implementation blueprints for the iFightDepression® tool.

### Selected countries

Three countries, Germany, Poland, and Spain, were selected as sites for this qualitative implementation mapping project.

Germany was purposefully selected because it was the first country to implement the iFD tool and accompanying awareness website. The program has been available nationwide since 2016, leading to substantial experience with both patient use and the number of trained user guides providing guidance.

Poland was chosen as a newer adopter of the guided iCBT version of the iFD tool. Implementation began in 2021, and the tool has been available nationwide since 2022.

Spain has implemented the iFD tool nationwide since 2018. Current efforts focus on promoting uptake in new regions and supporting sustainable expansion of the Spanish-language version.

### Project aims and objectives

The overall aim of this study is to address the gap between the demonstrated efficacy of the iFD tool for depression and its uptake and sustained use in routine clinical practice by identifying key barriers and facilitators across three countries.

The two primary objectives are:

1. To explore trained user guides’ experiences and perceptions of contextual factors (barriers and facilitators) influencing implementation, uptake (usage), and patient adherence to the iFD tool.
2. To develop theory-informed implementation blueprints, including a generic blueprint and country-specific versions for Germany, Poland, and Spain.

### Characteristics of study participants

Characteristics of participating user guides include country of practice (Germany, Poland, or Spain), professional role (e.g., family physician, psychiatrists or psychotherapists), years of professional experience, years of experience as an iFD user guide, and the number of patients guided through the tool.

### Outcomes

Primary outcomes focus on trained user guides perspectives regarding their experiences with the iFD tool. These include perceived barriers and facilitators to patient uptake and adherence, user guides’ experiences of supporting patients through the tool (e.g., content, usability, guidance process), and country-specific contextual factors such as organizational, cultural, or system-level influences. The study will also examine factors aligned with the CFIR domains that may inform future implementation strategies.

### Method

**Fig 1:**
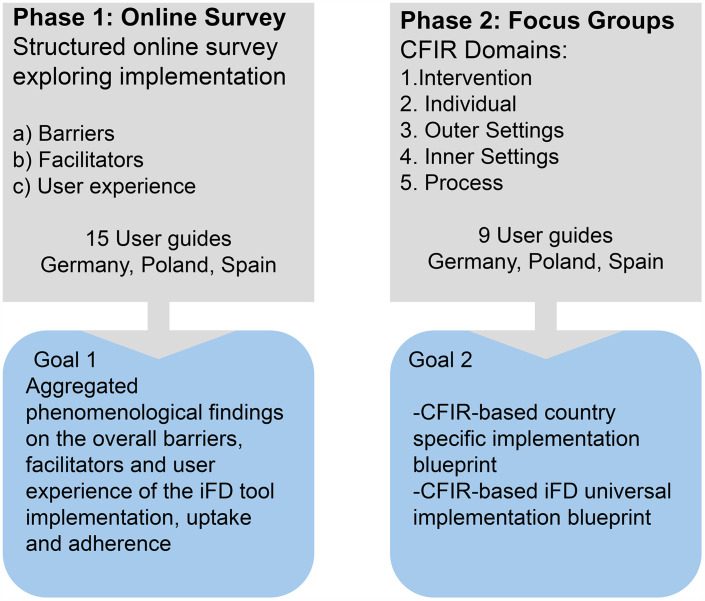
Methodological approach.

This study employs a two-phase qualitative design to capture trained user guides perspectives on their experiences with the iFD tool and to examine barriers and facilitators influencing uptake and adherence across countries. This approach enables a comprehensive understanding of overall user experiences, as well as tool-specific and country-specific factors influencing iFD use among referred patients. This includes both those patients who initiate the internet-based depression programme (uptake) and those who continue with or discontinue early from the treatment process (adherence). Together, the two phases support both exploratory inquiry and descriptive analysis. Fig 1 illustrates the two-phase methodological approach used in this study, combining an online survey and focus groups conducted across three countries.

Phase 1 applies Interpretative Phenomenological Analysis (IPA) to explore lived experiences in depth. IPA was selected for its idiographic orientation, which supports detailed examination of individual experiences within small, purposively selected samples. This approach is particularly suited to inductive, bottom-up inquiry, prioritising the richness and depth of participants accounts over sample size and enabling nuanced, interpretative engagement with their experiences [25,26].

Phase 2 employs template analysis to identify patterns across countries and to examine how contextual factors influence uptake and adherence. This analytic approach aligns with a qualitative paradigm that conceptualises knowledge as constructed and context-dependent [27–29]. A reflective analytic process informed by reflexive thematic analysis enables flexible, in-depth interpretation across the dataset and within country-specific groups, while maintaining attention to researcher reflexivity and supporting mapping of findings to CFIR implementation domains [28–31].

### Phase 1 – Data Collection

Phase 1 data will be collected using a semi-structured online survey designed to explore six pre-defined domains: motivation and role, experience with the iFD tool, barriers to adherence and uptake, facilitators of adherence and uptake, support and resources, and patient interaction and feedback. Within each domain, structured open-ended questions prompt participants to reflect on or describe their personal experiences related to specific aspects of iFD implementation. The survey interview guide was developed collaboratively by two researchers (MP and MZ) and subsequently pilot-tested by TR. Qualitative survey methodology was selected to facilitate open-ended responses and generate rich textual data beyond that captured by structured quantitative questionnaires [30]. The English version of the online survey interview schedule is provided in the Supporting Information (See S1 Appendix).

### Phase 2 – Data Collection

Phase 2 data will be collected through country-specific online focus groups guided by the Consolidated Framework for Implementation Research (CFIR). The focus group interview guides were developed by adapting CFIR open-ended question examples to align with the study’s aims and local implementation contexts. These guides were developed collaboratively by two researchers (MP and MZ) and pilot-tested by TR and CA to assess clarity, flow, and feasibility. Participants will take part in one of three CFIR-based focus groups to further explore their experiences, validate emerging findings from Phase 1, and identify contextual factors influencing iFD uptake and adherence. The English version of the focus group interview guide is provided in the Supporting Information (See S2 Appendix).

In summary, data will be collected from trained iFD user guides in Germany, Poland, and Spain through two methods: (1) individual completion of online semi-structured surveys by approximately 12–15 user guides, and (2) participation in country-specific online focus groups with three participants per country (total *N* = 9). Surveys will be administered via the Qualtrics platform and focus group discussions will be conducted using Zoom.

### Ethical approval

Ethical approval for this study was obtained from the Ethics Commission of the Faculty of Medicine, Goethe University, Frankfurt, Germany (reference number 2024−1937) on February 14, 2025.

### Data analysis

Phase 1: Data will be analyzed using IPA, following the broad analytical levels of analysis described by Smith and colleagues (2009), to identify and generate emergent themes on user experience, barriers, and facilitators. If the initial data lack sufficient idiographic depth, a more descriptive qualitative approach (e.g., reflexive thematic analysis) will be applied.

**Fig 2:**
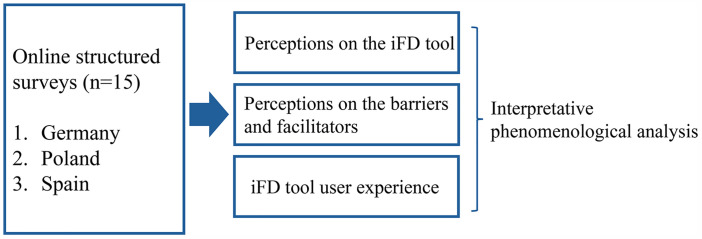
Data collection plan and analysis structure for Phase 1.

Fig 2 illustrates the Phase 1 analytic framework, showing how data from online structured surveys will be analysed using interpretative phenomenological analysis to examine perceptions of the iFD tool, barriers and facilitators, and user experience across participating countries.

Phase 2: Focus group data will be analysed using a customized version of the CFIR coding guidelines to code qualitative data from the focus group interviews using a template. The initial template will be deductively informed by CFIR domains (e.g., Intervention Characteristics, Outer/Inner Setting, Characteristics of Individuals, Process), while remaining open to inductive refinement. This template will help us to structure the qualitative data analysis using a deductive–inductive template analysis approach [27].

Two researchers will independently code a subset of transcripts in MAXQDA, NVivo, RQDA, or Excel to develop preliminary themes. They will then meet to agree on an initial template, including definitions, inclusion/exclusion criteria, and exemplar quotations which will be iteratively applied to the remaining data. The template will be modified as needed to incorporate emerging themes or to redefine, merge, or expand existing codes. The final template will integrate deductive CFIR-informed insights with inductive interpretations.

To allow for both within- and cross-country comparisons, a separate template will be developed for each country. This design enables the identification of shared versus context-specific implementation factors.

Focus group discussions will be transcribed using an AI transcription tool (e.g., TurboScribe), validated by the main researcher, and labelled using a consistent scheme (e.g., FG01, FP02, FS03). Findings from Phase 1 interviews (IPA) will inform the interpretation of the template analyses, helping to distinguish tool-specific and country-specific barriers and facilitators from overarching perceptions of the iCBT tools.

**Fig 3:**
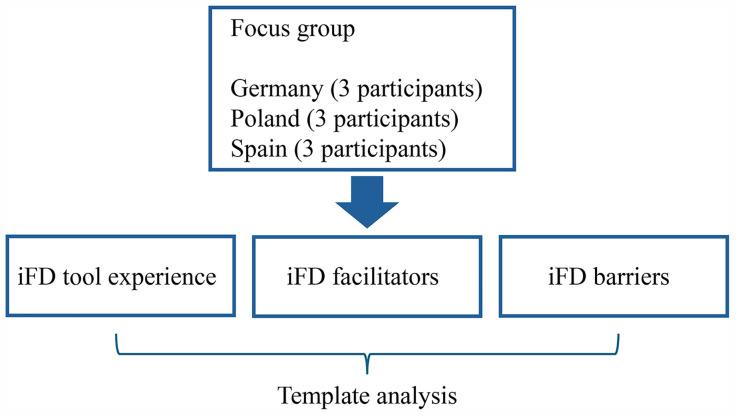
Data collection planned analysis structure for Phase 2.

Fig 3 illustrates the Phase 2 analytic approach, showing how country-specific focus group data will be analysed using template analysis to examine experiences with the iFD tool, as well as perceived facilitators and barriers.

Credibility and trustworthiness will be strengthened through triangulation, member reflections, thick description, and multivocality, consistent with qualitative rigor criteria [32].

### Study Participants

#### Inclusion Criteria.

Participants must have completed the mandatory iFD user guide training module and have guided at least one patient through the iFD tool.

#### Exclusion Criteria.

Individuals who have not completed the required user guide training or who have not yet guided a patient through the tool will be excluded.

#### Number of Study Participants.

The study will include 12–15 trained iFD user guides for the online qualitative survey and nine trained user guides for the focus groups (three user guides per country).

#### Sampling.

Consistent with qualitative research recommendations, a sample of approximately 12 interviews is expected to reach thematic saturation for focused research questions involving relatively homogeneous participant groups [33,34]. IPA studies typically use small sample sizes to enable in-depth idiographic analysis. As Smith and colleagues (2009) [25] note, “the aim is to find a reasonably homogeneous sample so that, within the sample, we can examine convergence and divergence in some detail” (page.3).

### Participant recruitment

National coordinators of the iFD tool in Germany, Poland, and Spain will identify potential participants who meet the study selection criteria. E-flyers will be shared with eligible user guides and will include a QR code linking to the SoSci Survey platform, where full study information will be provided. After reviewing the study information, potential participants may choose to participate in either the online survey or the focus group discussions.

To ensure the successful recruitment and retention of iFD tool user guides across Germany, Poland, and Spain, a participant compensation strategy in the total value of 40 euros for each participant will be implemented for the focus group interviews. Furthermore, because Phase 2 focus groups rely on a small sample of only three participants per country, compensation serves as a vital tool to mitigate recruitment risks and prevent attrition that could otherwise hinder the study’s ability to reach thematic saturation. If additional compensation is required to provide incentives for the online survey, the study team will explore supplementary funding opportunities. This proactive approach is expected to facilitate the inclusion of a broader range of professional perspectives, including those from participants who might otherwise be unable to commit time to the study.

Recruitment will begin immediately after publication of this study protocol. The estimated project end date is December 2026.

### Informed consent

Participation is voluntary. Written informed consent will be obtained before data collection begins. Consent materials will describe study procedures; risks and benefits; the right to withdraw at any time; privacy and data protection; anonymity and confidentiality; and the right to request removal of personal data. The participant information sheet and consent form will be available in English, German, Polish, and Spanish.

User guides will have access to country-specific language versions of the online survey and focus group questions. National iFD tool coordinators will facilitate the focus groups and support translation to minimize language barriers. The voluntary consent of participants is the legal basis for data processing under the German Data Protection Regulations (GDPR) (Article 6(1)(a)).

### Data management

Audio recordings and transcripts will be stored on a password-protected server at the Research Center of the German Foundation for Depression and Suicide Prevention, with audio files deleted within three months following verification of transcript accuracy. All participants will provide written informed consent, and no patient-level data will be collected. All data will be de-identified, with no linking documents (e.g., master lists with pseudonyms) retained after data collection is complete. Findings will be reported in aggregate form wherever possible, and de-identified participant quotations may be included as illustrative examples and attributed to pseudonyms to preserve anonymity. Participants may request withdrawal or deletion of their data at any point during or after participation, and their rights concerning data access, correction, deletion, and restrictions on processing will be clearly communicated in accordance with GDPR requirements.

## Discussion

This multi-country qualitative study is designed to address the persistent gap between the demonstrated efficacy of guided internet-based interventions for depression and their uptake and sustained use in routine clinical practice. Although the iFD tool is supported by a growing evidence base [10,35,36], its integration into everyday care remains limited. By examining how trained user guides experience the tool in clinical practice and how they perceive patient motivations and challenges, this study aims to generate insights that can inform more effective implementation strategies.

The study is expected to highlight the central role of user guides in shaping patient engagement with the iFD tool, particularly during the early stages of uptake and adherence. Exploring how user guides interpret patient readiness, motivation, and responsiveness to guidance may provide valuable insight into how referral practices, ongoing support, and continuation of use are influenced in real-world settings. These perspectives are anticipated to extend existing understandings of digital intervention implementation by highlighting the relational and professional dimensions of guided care.

Variation across countries is likely to reveal both shared and context-specific influences on implementation. Differences in health-care systems, organizational structures, and stages of national implementation may shape how barriers and facilitators are experienced, underscoring the importance of contextual sensitivity when implementing guided digital interventions at scale [37]. Rather than assuming uniform pathways to adoption, the study seeks to clarify how local conditions interact with tool characteristics and professional roles.

Several methodological considerations should be acknowledged in interpreting the anticipated findings. As a qualitative study, the focus is on depth and contextual richness rather than statistical generalizability. However, integrating perspectives across study phases and countries, and situating findings within an established implementation framework, is expected to enhance interpretability and analytic coherence. While online data collection increases accessibility, it may limit some aspects of interpersonal interaction typically achieved through in-person qualitative methods.

Ultimately, this study aims to move beyond identifying barriers and facilitators toward generating practical implementation insights. By integrating country-specific considerations with cross-cutting themes, the findings are intended to inform the development of tailored and sustainable implementation strategies for the iFD tool. More broadly, the study seeks to contribute to the growing literature on guided iCBT by offering transferable insights into how evidence-based digital interventions can be better aligned with routine clinical practice, helping to narrow the enduring gap between research evidence and real-world use.

## Supporting information

S1 Appendix English version of the online survey interview schedule. (PDF)

S2 Appendix English version of the focus group interview guide. (PDF)
