# Supplementary material for: Study Protocol: It is time to dig deeper: A cross-country implementation mapping study of the iFightDepression® (online self-management) tool
Source: PLoS One. 2026 Mar 12;21(3):e0343982. doi: 10.1371/journal.pone.0343982 (PMC12981504; doi:10.1371/journal.pone.0343982)
Supplement: S1 Appendix — (PDF) [file pone.0343982.s001.pdf]

IPA: Individual Online Interviews (Perceived Barriers/Facilitators)

Focus: user experience and perceived barriers and facilitators influencing adherence and uptake of the iFightDepression®-tool

1. Motivation and Role:

- Please reflect on your motivations for becoming an iFD user guide.
- How would you describe your user guide role compared to your professional role?

2. Experience with the iFD Tool in one's practice:

- How does the iFD tool integrate into your existing clinical or professional practice?

3. **Barriers to Adherence and Uptake**:

- What challenges/barriers, if any, have you encountered when guiding patients to use the iFD tool?
    prompts: technical issues, organizational barriers…
- What features or functionalities would enhance iFD usefulness for individuals with depression?
- Why, do you think, patients may drop out or choose not to use the iFD tool?
- Are there particular times during the treatment when patients are more likely to stop using the iFD tool? If so, why do you think this happens?

4. **Facilitators to Adherence and Uptake**:

- What factors/conditions have facilitated the successful use of the iFD tool among your patients? (could you elaborate how?)
- How do you encourage patients to adhere to the iFD tool?

5. Support and Resources:

- What additional support or resources do you believe would help you better guide patients using the iFD tool?

6. Patient Interaction and Feedback:

- How do patients typically respond to the iFD tool?
- What is your experience with receiving these reports from patients?
- Can you tell me about any positive outcomes/success stories from using the iFD tool with your patients?
