# Supplementary material for: Study Protocol: It is time to dig deeper: A cross-country implementation mapping study of the iFightDepression® (online self-management) tool
Source: PLoS One. 2026 Mar 12;21(3):e0343982. doi: 10.1371/journal.pone.0343982 (PMC12981504; doi:10.1371/journal.pone.0343982)
Supplement: S2 Appendix — (PDF) [file pone.0343982.s002.pdf]

	iFD tool questions
1.	How credible and trustworthy do you think the iFD tool is?<br>(prompt in case one dimension is missing)
2.	Could you elaborate on some information or evidence that shows the effectiveness of the iFD tool in your work?
3.	Can you describe your experience or any challenges you've encountered in implementing the iFD tool?
4.	How user-friendly is the iFD tool?
5.	Considering that the iFD tool is free of charge, how did that influence your decision to use it?
6.	Tell me about any major incidents that affected how well the tool can be used in your practice?<br>Prompt: Was there any unanticipated event/disruption in using the iFDtool?
7.	How do you perceive sociocultural beliefs to affect the public attitudes on using the iFD tool?
8.	Tell me about country specific circumstances that support the use of the iFD tool?
9.	How would you describe local networks or connections with other healthcare professionals who use the iFD tool?
10.	How do policies, regulations, and clinical practice guidelines affect how you use the iFD tool? (defining clinical practice guidelines for the user if needed)
11.	What external pressures (e.g., societal, market, performance measurements, peer pressure, or competing organizations) are influencing the implementation of the iFD tool?
12.	Tell me about any external factors, such as media campaigns, advocacy groups, or social movements, that are supporting the use of the iFD tool?
13.	How would you describe your work environment in supporting your use of the iFD tool? Prompts: communication, roles and hierarchy
14.	Please describe any tensions about using the iFD tool in your workplace?<br>Prompt: What do you think needs to change?
15.	How important is the use of the iFD tool compared to other projects in your work?<br>Prompt: Why is the iFD tool more important than other interventions?
16.	Are there any (local) incentives or rewards that support the use of the iFD tool in your work?
17.	Considering your work organization goals related to depression and suicide, how would you describe the iFD tool's alignment with those goals?
18.	What resources do you have that facilitate the use of the iFD tool?

19.	Could you describe how easy it is to do the user guide training?<br>Prompt: How effective was the training to become an iFD tool user guide?<br>How much time did it take to do the training?
20.	How is your leadership team supporting you?
21.	Are there any other important individuals who support you in using the iFD tool?
22.	Could you tell me if the use of the iFD tool brings or gives you a personal fulfillment in your job?
23.	Do you feel you have the necessary skills and knowledge to provide guidance to patients using the iFD tool?
24.	Do you have enough time and control over your work schedule to fulfil your role as iFD tool user guide?
25.	How committed are you to use (or to continue using) the iFD tool in your practice?
26.	How do you assess the needs and preferences of your patients when deciding on using the iFD tool?
27.	Please describe what are the priorities, preferences and potential needs of the iFD users that guide your use of the tool?
28.	28a: How would you reflect on the barriers for using the iFD tool?<br>Prompt: What do you think are the most important barriers in using the iFD tool<br>28b: How would you reflect on the facilitators for using the iFD tool?<br>Prompt: What do you think are the most important facilitators in using the iFD tool?
29.	How did you address the aforementioned barriers and facilitators to better apply the iFD tool to ensure it aligns with your work context?
30.	How do you encourage the user participation in the iFD tool?
31.	How do you evaluate, if at all, the use of the iFD tool?
32.	Is there anything else you would like to add about your experience with the iFD tool that we have not covered?
